# Doing More Concrete Work

**Published:** 1916-09

**Authors:** 


### Doing More Concrete Work.

That the Texas Medical Association should do some more concrete work than it has heretofore done is admitted by everyone who has studied the organization closely. If it would have an influence that will really count for something, it must become more than a mutual admiration society with annual meetings. It must do more than prate about ethical codes and have a greater ambition than merely to assemble numbers, and have a cash balance in the treasury after the yearly expenses are paid. It must have something more to commend it than dignity. Physicians individually learned this several years ago and most of them removed their side whiskers, those impressive emblems of professional character. Now the muchly bewhiskered man is in danger of being regarded as a quack. The Medical Association should follow the example set by the individuals who compose it, and lay aside some of its dignified bearing and get down to more practical things that will endear itself to the people and make it of genuine, practical helpfulness. This is decidedly an era of constructive and creative work, and no organization can thrive long on its past record.

This is not a criticism of the Texas Medical Association for what it has done, but is merely a suggestion that it should start out to do something greater and better. Public health conditions should be improved all through the State, and unnecessary diseases should be wiped out of the land. This can be done if only the organized physicians will throw themselves boldly and untiringly into the work. If really interested in serving humanity, as they claim to be, and as most of them are, they will go about this unselfishly. All that is needed to bring this about is the suggestion and the leadership. The great majority of the membership of the medical profession want to see all unnecessary diseases eliminated, but they feel that they cannot do it without proper organized effort. The State Health Boards are doing what they can, but they can't succeed unless they have the help of the physicians. Officers are in some places attempting to enforce health rules and laws, but they have to depend largely on public sentiment, and that must be created by the physicians,—not by the individual physician here and there, but by the organized profession.

The Texas Medical Association can well afford to have committees from its membership look into conditions in every county, and demand that where necessary they be improved. We hear much said in a general way about the unnecessary confinement of the insane in the county jails, and about the unwholesome conditions under which these unfortunates are confined, but who ever hears any definite statements as to conditions in any specific jail? Occasionally there are rumors afloat that perhaps the treatment of the insane in all the asylums is not just what it should be, but when has there ever been any expert professional study of asylum management, except on the part of those who are in charge of these institutions? They may be conducted along the most approved plans, in keeping with the progress of medical science, and if they are they at least deserve some professional recognition of their achievements; on the other hand, they might be a hundred years behind in their methods so far as the organized medical profession seems to know or care. Why should not the Texas Medical Association co-operate with the asylum managements in their efforts to help a great unfortunate class? No doubt such co-operation would be welcomed, and it would show that the Association is interested in some big, practical problems.

There are a hundred other ways in which the Association can get about doing something that will direct attention to it and will make it a strong power in the State. It should not be content to rest on what it has done, neither should it be satisfied just to hold its meetings and discuss the things that will only aid its members in some way, but it should take up such practical work as will entitle it to standing as a great altruistic organization, interested in the general good of the people. There need be no fear, and there is none on the part of the better class of physicians, that the public health will ever be so effectually conserved that the services of physicians will be no longer in demand. There may in time come about radical changes in the professional duties of those who minister to the sick and afflicted, but when such changes do come (if they do), the progressive men, who sympathetically have studied the needs of mankind, and not those who have been obstacles in the way of progress, will be the men in greatest demand.

